# Speech Changes After Second-Side Magnetic Resonance-Guided Focused Ultrasound (MRgFUS) Thalamotomy: Is Intraoperative Speech Evaluation Associated With Speech Outcomes?

**DOI:** 10.7759/cureus.112488

**Published:** 2026-07-11

**Authors:** Trevor A Leon, Kristina Haker, Daniel Gaudin

**Affiliations:** 1 Boonshoft School of Medicine, Wright State University, Dayton, USA; 2 Department of Neurosurgery, Clinical Neuroscience Institute, Premier Health, Dayton, USA

**Keywords:** bilateral, essential tremor, focused ultrasound, language, mrgfus, speech, thalamotomy, ventral intermediate nucleus

## Abstract

Introduction

Staged bilateral magnetic resonance-guided focused ultrasound (MRgFUS) thalamotomy is an emerging option for the treatment of bilateral essential tremor (ET). However, there is historical concern that permanent bilateral thalamic lesioning leads to high rates of speech complications. This study evaluates bilateral MRgFUS thalamotomy of the ventral intermediate nucleus (VIM) for essential hand tremor, focusing on the impact of intraoperative speech monitoring during second-side procedures and its association with speech-related adverse effects (AEs).

Methods

A retrospective review was conducted, including 13 patients who underwent staged bilateral MRgFUS thalamotomy for medication-refractory hand tremor at Miami Valley Hospital between April 2020 and March 2023. Data collected included demographics, operative parameters, and AEs. Tremor outcomes and complications were assessed immediately following each procedure and at scheduled follow-up visits two weeks postoperatively. During the second-side procedure, a speech therapist conducted formal perioperative and intraoperative speech evaluations to identify deviations from baseline. Patients who reported speech changes at the two-week follow-up after the second-side procedure were contacted two years postoperatively for further assessment. Only descriptive statistics were utilized because of the small sample size.

Results

All 13 patients achieved near-complete bilateral tremor resolution. Following the first-side procedure, 23.1% of patients reported changes in speech. Additional AEs following the first-side procedure included lip numbness (30.8%), finger numbness (8.0%), and unsteady gait (8.0%). Nearly all resolved within two weeks. Following second-side treatment, immediate AEs included lip/tongue numbness (53.8%) and gait imbalance (8.0%). Only one patient (8.0%) reported an intraoperative speech change, and no patients deviated from baseline according to the formal intraoperative speech assessment. At the two-week follow-up, four patients (30.8%) reported taste disturbance, four (30.8%) reported lip/tongue numbness, three (23.1%) reported persistent subjective speech changes, one (8.0%) reported imbalance, and one (8.0%) reported bilateral leg weakness. Among the three patients reporting speech disturbance at the two-week follow-up, two reported complete resolution at the two-year follow-up, whereas one patient reported persistent mild slurring.

Conclusions

Staged bilateral MRgFUS VIM thalamotomy provided near-complete tremor control in all patients, with most AEs being transient or improving by short-term follow-up. Importantly, speech complications following the second-side procedure were lower than those historically reported with bilateral lesioning. Intraoperative deficits following second-side thalamotomy with intraoperative speech evaluation were limited and included only one long-term persistent case. This patient with persistent deficits was also the only patient to experience a transient speech change during the initial procedure. Incorporating intraoperative speech evaluation during second-side treatment may aid in minimizing language difficulties without compromising optimal targeting for maximal tremor control.

## Introduction

Essential tremor (ET) is one of the most common neurological conditions worldwide. A recent meta-analysis estimated that 24.91 million people are affected by this condition globally [1]. However, many population-based studies have shown that only a very small proportion of ET cases are reported, leading to a gross underestimation of the actual global prevalence [2]. Although ET does not change life expectancy, it can significantly affect a patient’s quality of life. The tremor typically begins unilaterally but frequently progresses to involve the bilateral extremities within a matter of years, leading to significant difficulty accomplishing daily tasks [3]. The pathophysiology of ET is not completely understood; however, damage to the cerebellum and the cerebello-thalamic-cortical circuit has been commonly observed in these patients [4]. Due to possible disinhibition of deep cerebellar neurons, increased activity throughout the cerebello-thalamic-cortical tract has been shown to lead to oscillating tremor [5]. Because of the role of the ventral intermediate nucleus (VIM) in the cerebello-thalamic-cortical circuit, it has become the primary target for ablative and neuromodulatory procedures aimed at treating ET.

Magnetic resonance-guided focused ultrasound (MRgFUS) uses transcranial focused ultrasound to create an ablation in the VIM of the thalamus for patients with ET. The effects are instantaneous, allowing for real-time adjustments based on changes in clinical response [6]. Since patients remain awake, clinical staff are able to directly monitor functional changes throughout the procedure. Unlike deep brain stimulation (DBS), MRgFUS requires no skin incisions or penetration into the brain parenchyma and is frequently performed in an outpatient setting [7]. Unilateral MRgFUS ablations have been used to address medically refractory ET on the contralateral side. Many studies have investigated the safety of bilateral thalamotomy procedures using various ablative techniques, including thermocoagulation, radiofrequency, and Gamma Knife [8]. Permanent bilateral thalamotomy has raised significant concern because decades of research have demonstrated unsatisfactory complication rates, including sensory changes, ataxia, gait disturbances, and, most critically, changes in speech and language. Speech and language are of particular concern because, unlike sensory or coordination changes, they directly affect a patient’s ability to communicate and may significantly impair quality of life.

A recent meta-analysis reported that among patients who underwent ablative thalamotomy using one of the aforementioned techniques, 19.8% experienced speech problems. Among those treated unilaterally, 15% had speech issues, whereas 40.6% of those treated bilaterally were affected. Additionally, speech difficulty was two to three times more common after left-sided procedures. Among patients who underwent DBS, 19.4% experienced speech problems [9]. DBS is a nonpermanent and reversible method of bilateral thalamic neuromodulation that is considered the current gold standard treatment for ET. With the more recent development of noninvasive techniques such as MRgFUS, patients are able to achieve significant tremor control without the need for an implantable device, which is often placed in multistage procedures and requires ongoing maintenance and eventual replacement. Additionally, MRgFUS is associated with lower risks of infection and bleeding and often does not require an inpatient hospital stay. This promising technique currently lacks sufficient data to comprehensively evaluate the safety and efficacy of bilateral thalamotomy for ET, especially with respect to speech and language.

## Materials and methods

Study design

In this single-institution study, 13 patients who underwent staged bilateral MRgFUS thalamotomy of the VIM for the treatment of bilateral essential hand tremor between April 2020 and March 2023 were identified. To assess the safety and efficacy of second-side MRgFUS VIM thalamotomy, patients’ medical records and Insightec operative reports (Haifa, Israel) were reviewed retrospectively. For each patient, the following data were collected: skull density ratio (SDR), operation dates and side, anterior commissure-posterior commissure target coordinates, number of sonications, and average and peak ablation temperatures. Additionally, intraoperative and postoperative notes were reviewed to determine procedural outcomes and any adverse effects (AEs) following the procedure.

Patient selection criteria

Our study followed the patient selection criteria and contraindications for the MRgFUS procedure outlined in the Insightec Information for Prescribers document [10]. Patients included men and women aged 22 years or older with a confirmed diagnosis of ET refractory to propranolol and/or primidone.

Exclusion criteria included patients with standard contraindications to MRI, such as non-MRI-compatible implanted metallic devices, including cardiac pacemakers; patients with body size limitations for MRI; or patients with allergies to MR contrast agents. Additional exclusion criteria included pregnancy; advanced kidney disease or dialysis; unstable cardiac status or severe hypertension; behaviors consistent with ethanol or substance abuse; a history of abnormal bleeding, hemorrhage, and/or coagulopathy; receipt of anticoagulants or drugs known to increase the risk of hemorrhage within one month of the focused ultrasound procedure; cerebrovascular disease; brain tumors; inability or unwillingness to tolerate the prolonged stationary position required for treatment; unstable psychiatric disease, uncontrolled depressive symptoms, psychosis, delusions, hallucinations, or suicidal ideation; and an overall SDR of less than 0.40 (± 0.05), calculated from the initial screening CT.

For staged bilateral ET treatment, patients who experienced moderate to severe clinically significant dysphagia, abnormal speech function, or gait abnormalities following the initial unilateral thalamotomy procedure were excluded from second-side treatment. For all patients, the first-side procedure was completed at least nine months before initiation of the second-side procedure.

MRgFUS procedure

The device utilized in this study was the Insightec Exablate Model 4000. Standard treatment procedures were followed according to those outlined in the Insightec Exablate Model 4000 Operator Manual [11].

On the day of the procedure, a stereotactic frame was placed on the patient’s head under local anesthesia. A rubber membrane was affixed to the scalp as the frame was secured to the ultrasound transducer. The membrane was filled with cold, degassed water and grossly aligned with the left thalamic target. Reference images were acquired using a 1.5-T MRI system, including a volumetric sequence, and were reconstructed in planes orthogonal to the anterior and posterior commissures. The appropriate thalamic target was then planned.

Targeting was performed using stereotactic coordinates for the VIM on the appropriate side. These coordinates were generally determined by measuring 11 mm lateral to the wall of the third ventricle, 1.5-2.0 mm superior to the intercommissural plane, and locating a point anterior to the posterior commissure corresponding to 25% of the intercommissural distance. Appropriate pass regions were designated for intracranial calcifications, frontal air sinuses, and membrane folds. A movement-detection algorithm was applied to ensure stability before therapeutic sonication.

A series of alignment sonications, with peak temperatures up to 50 °C, was performed to ensure that the natural focus of the transducer matched the two-dimensional terminal plane of the MRI. Sequential target sonications at lower peak temperatures (51-55 °C) were performed, with clinical examinations conducted between each sonication to confirm target location and treatment effect. Final therapeutic sonications were initiated after satisfactory alignment had been achieved, with peak temperatures between 55 and 63 °C. Once upper-extremity tremor was significantly improved, two additional therapeutic lesions were created 0.5 mm and 1.0 mm above the target. At the completion of the procedure, patients self-reported any sensations that deviated from their baseline, and the stereotactic frame was removed.

AEs and follow-up

For both procedures, a two-week follow-up was conducted to assess treatment outcomes and residual AEs. AEs were defined as deviations from the patient’s preoperative baseline and were categorized according to subjective patient reports obtained immediately after each procedure, at the two-week follow-up, and at the two-year follow-up. The two-year follow-up was conducted by telephone only for patients who reported speech changes at the two-week follow-up after the second-side procedure. This follow-up was performed solely to determine whether self-reported speech changes had persisted or resolved. Accordingly, the two-year follow-up was limited to the three patients who reported speech-related AEs at the two-week follow-up.

AEs were considered transient if they occurred immediately after the procedure but were no longer reported at the two-week follow-up. AEs were classified as persistent if they were present immediately after the procedure and remained present at the two-week follow-up. Same-day headache and nausea were not reported because they are common, expected transient effects following MRgFUS thalamotomy and were not the focus of this study. All follow-up data were collected retrospectively through chart review.

Intraoperative speech evaluation

For second-side procedures, a speech-language pathologist was present to monitor treatment-related changes in communication, cognition, oral sensation, and swallowing. Patients underwent approximately 30-minute evaluations before and after the procedure to establish baseline performance and identify any acute postoperative changes. The assessment included receptive and expressive language, voice and speech production, respiratory support and coordination for speech, oral-motor function, cognitive-linguistic performance, and swallowing safety.

Receptive and expressive language abilities were evaluated through informal observation during the patient interview and communication-focused case history. Auditory comprehension was assessed by evaluating the patient’s ability to understand questions and follow commands. Expressive language was evaluated during spontaneous conversation, with attention to sentence completeness, organization of thought, fluency, and evidence of word-retrieval difficulty. A brief confrontational naming task consisting of approximately 10 pictured objects was also administered as a rapid screen of lexical retrieval.

Voice, speech, respiratory support, and speech-breathing coordination were assessed using a dysarthria profile. Respiratory assessment included speaking volume and the appropriateness of the number of words or syllables produced per breath. Phonatory assessment included the ability to sustain phonation, vary vocal pitch, and maintain appropriate resonance, including evaluation for hypernasality or hyponasality. Speech production was assessed through repetition of single words and phrases, with attention to articulatory precision and overall intelligibility. Articulation and intelligibility were also monitored during spontaneous conversation.

An oral-motor examination was performed to assess the structure and function of the lips, tongue, and palate, including range of motion, speed, symmetry, coordination, and accuracy. Facial symmetry, dentition, oral mucosa, oral sensation, and labial, lingual, and velar function were documented. Cognitive-linguistic assessment included attention, orientation, and immediate verbal memory. Immediate recall was evaluated using the Story Memory subtest of the Repeatable Battery for the Assessment of Neuropsychological Status, during which patients listened to a short narrative passage and reproduced as many details as possible. Sustained and selective attention were assessed throughout testing and conversation, including the patient’s ability to maintain task performance in the presence of environmental distractions. Basic orientation questions were used to assess orientation to person, place, time, and situation.

The Stroop test was administered at three intraoperative time points as a brief measure of attention, processing speed, executive function, and cognitive flexibility. Performance was evaluated by comparing the number of errors made while reading words or identifying colors with each patient’s baseline performance [12]. Because repeated exposure could result in practice-related improvement, intraoperative and postoperative performance was interpreted primarily as evidence of stability or deterioration from baseline rather than as an independent measure of cognitive improvement.

During the MRI-guided procedure, the speech-language pathologist entered the procedural area with the treatment team to conduct brief interval assessments. Monitoring focused on changes in speech production, intelligibility, communication ability, and patient-reported alterations in facial, oral, or lingual sensation. When procedural time permitted, patients were also asked questions regarding the previously presented narrative or Stroop task. Following the procedure, swallowing function was observed during oral intake of a snack, particularly in patients reporting changes in oral sensation. Most patients demonstrated no observable changes in communication performance.

Patients additionally self-reported perceived changes in speech, language, oral sensation, and related functions immediately after treatment, at the two-week postoperative follow-up, and at the two-year follow-up when longitudinal data were available.

Statistical analysis

Because of the small sample size (N = 13), only descriptive statistics were used to analyze the data. No comparative or longitudinal inferential statistical analyses were performed because the study focused on reporting frequencies and ranges. All percentages were calculated based on the total cohort of 13 patients.

Ethical approval

This study was approved on August 22, 2024, by the Wright State University Institutional Review Board (IRB) as a retrospective chart review covering procedures performed between 2020 and 2023 (IRB-2024-590). All study procedures were performed in compliance with relevant laws and institutional guidelines. Informed consent was waived by the IRB, and the privacy rights of human subjects were maintained throughout the study.

The data supporting the findings of this study are available from the corresponding author upon reasonable request.

## Results

Patient demographics 

Patients’ ages at the time of the initial thalamotomy ranged from 63 to 76 years, with a median age of 68 years. At the time of the second thalamotomy, ages ranged from 64 to 77 years, with a median age of 69 years. Of the 13 patients, two (15.4%) were female, and 11 (84.6%) were male. The median duration of ET before the initial thalamotomy was 24 years. The median interval between the initial and second-side thalamotomies was 15.8 months. Of the study cohort, 10 (76.9%) patients were right-handed and three (23.1%) were left-handed. Except for one patient, all patients were treated on their dominant side first. The single patient who underwent treatment on the nondominant side first had a more severe symptom burden in the nondominant hand, warranting earlier treatment. Table 1 outlines the baseline demographic characteristics of the study population.

**Table 1:** Baseline demographic characteristics

Characteristic	Value
Median age at first-side procedure	68 years (range, 63-76)
Median age at second-side procedure	69 years (range, 64-77)
Median duration between diagnosis and first procedure	24 years
Median duration between first and second procedures	15.8 months
Handedness	Right: 10 (76.9%); left: 3 (23.1%)
Sex	Male: 11 (84.6%); female: 2 (15.4%)

Tremor outcomes and AEs

Of the patients reviewed, all 13 experienced near-complete hand tremor resolution following both the initial unilateral and second-side VIM thalamotomy. Patients evaluated their tremor status immediately following each procedure and reported a subjective percentage of tremor resolution.

The AEs assessed following the first-side procedure were typical and expected based on the prior literature. At the initial postoperative evaluation, there were four reported cases of lip numbness (30.8%), one reported case of finger numbness (8.0%), and one reported case of unsteady gait (8.0%). On the initial speech evaluation, three patients (23.1%) experienced transient speech difficulty. At the two-week follow-up, most AEs had resolved, with one patient (8.0%) reporting lip and thumb numbness and one patient (8.0%) reporting imbalance. Table 2 presents these findings in greater detail.

**Table 2:** Results of first-side VIM thalamotomy Hand tremor resolution was determined subjectively by the patient following the procedure. All AEs marked with an asterisk (*) were transient and resolved completely within two weeks postoperatively. Bold text indicates the speech changes of interest. AE, adverse effect; VIM, ventral intermediate nucleus

Patient no.	Side	Hand tremor resolution	Initial AEs	Speech changes
1	R	100%	-	-
2	L	100%	-	-
3	L	90%	-	-
4	L	98%	Lip numbness*	-
5	L	95%	-	-
6	R	100%	Lip numbness*; finger tingling*	-
7	L	100%	Lip numbness*	-
8	L	90%	Lip numbness*; unsteady gait*	Speech difficulty; slurring; increased pitch
9	L	100%	-	-
10	R	-	-	-
11	L	95%	-	-
12	L	100%	-	Speech difficulty*; hiccups*
13	L	100%	-	Slurring*; changes in “s” sound*

Same-day headache and nausea were not considered AEs in this study because they are common, expected, and transient occurrences following these procedures.

From the initial evaluation following the second-side procedure, seven patients experienced lip/tongue numbness (53.8%), and one patient reported minor gait imbalance (8.0%). Only one patient (8.0%) experienced minor intraoperative speech changes; however, upon formal evaluation by a speech-language pathologist, no patients met the criteria for significant deviation from baseline. As such, the data presented in the tables are based on patient self-report.

At the two-week follow-up, four patients reported taste deficits (30.8%), four reported lip or tongue numbness (30.8%), three reported speech changes consistent with dysarthria (23.1%), one reported persistent imbalance (8.0%), and one reported bilateral leg weakness (8.0%). The single patient (Patient 8) who experienced initial speech changes also reported persistent slurring at the two-week follow-up. This patient was contacted two years postoperatively and reported no improvement in the slurring. The two additional patients (Patients 1 and 11) who reported speech changes were also contacted two years postoperatively and reported no speech or language deficits. Table 3 presents the results of the second-side procedure.

**Table 3:** Results of second-side VIM thalamotomy Hand tremor resolution was determined subjectively by the patient following the procedure. All AEs marked with an asterisk (*) were transient and resolved completely within two weeks postoperatively. Bold text indicates the speech changes of interest. Only speech changes were recorded in the two-week follow-up column. AE, adverse effect; VIM, ventral intermediate nucleus

Patient no.	Side	Intraoperative speech evaluation	Hand tremor resolution	Initial AEs	Speech changes	Two-week follow-up (speech)	Two-year follow-up (speech)
1	L	No change from baseline	100%	-	-	Hoarse voice; lower pitch	No deficits
2	R	No change from baseline	95%	-	-	-	-
3	R	No change from baseline	90%	-	-	-	-
4	R	No change from baseline	100%	Lip numbness*	-	-	-
5	R	No change from baseline	95%	Lip/tongue numbness	-	-	-
6	L	No change from baseline	99%	Lip numbness	-	-	-
7	R	No change from baseline	100%	-	-	-	-
8	R	No change from baseline	90%	Dizziness; imbalance	Increased pitch; “crackly” voice	Slurring	Slurring
9	R	No change from baseline	100%	-	-	-	-
10	L	No change from baseline	90%	-	-	-	-
11	R	No change from baseline	100%	Lip numbness*; imbalance*	-	Hoarse voice	No deficits
12	R	No change from baseline	90%	Lip numbness*	-	-	-
13	R	No change from baseline	85%	Lip/tongue numbness*	-	-	-

## Discussion

Bilateral MRgFUS thalamotomy presents a promising therapeutic option for treating bilateral ET of the hand, achieving near-complete tremor resolution in all patients in this study. A number of studies have investigated the safety and efficacy of bilateral MRgFUS thalamotomy. One case report described a 57-year-old man with medication-refractory ET who underwent bilateral MRgFUS thalamotomy. Tremor in both hands resolved following contralateral thalamotomy, and no serious complications were reported [13]. A two-center study showed that second-side intervention led to a statistically significant and clinically meaningful improvement in quality of life, as measured using ET-specific instruments. After the procedure, 70% of patients experienced adverse outcomes; however, these were no more frequent or severe than those observed after the first-side procedure. Additionally, all patients reported that the benefits of the procedure outweighed the drawbacks [7]. Other studies have concluded that bilateral MRgFUS thalamotomy is an effective treatment for bilateral ET, provided that the lesions are asymmetrical and the second procedure is performed at least one year after the first-side procedure [14,15]. Further studies focusing on speech changes are limited, and additional research is needed to establish its safety with respect to speech and language.

In our study, AEs increased following the second-side thalamotomy compared with the initial procedure; however, most AEs were transient or improved by the two-week follow-up. Importantly, all reported outcomes in this study were subjectively determined by the patients, except for the intraoperative speech evaluation conducted by a speech-language pathologist. Although these assessments lacked objective outcome measures, they allowed us to evaluate the impact of the procedure on quality of life from the patients’ perspectives, which is clinically meaningful in this setting. A meta-analysis by Alomar et al. reported a 35% increase in speech changes from the first-side procedure to the second-side procedure [9]. Interestingly, in our study, there was a decrease in initial speech changes following the second-side procedure (one patient) compared with the first-side procedure (three patients). We propose that the use of intraoperative speech evaluation may have contributed to this decrease. One possible explanation is that intraoperative speech evaluation allowed us to minimize speech difficulties in real time without compromising optimal targeting for near-complete tremor control. Based on our literature search, only one other group has utilized objective speech evaluation; however, it was performed three months postoperatively [16]. To the best of our knowledge, our study is the first to utilize objective intraoperative speech evaluation during second-side MRgFUS thalamotomy.

Although initial speech changes were limited to one patient, three patients reported persistent speech changes at the two-week follow-up. One possible explanation is that awareness of subtle deficits does not occur until patients have spent more time outside the hospital. During this period, patients have more opportunities to communicate in everyday settings, increasing the likelihood of recognizing subtle changes. Additionally, friends and family members may notice differences that the patients themselves had not recognized. Another possible contributing factor is lesion size and associated edema. Lesion volume and edema have been shown to reach their maximum on postoperative day 3, with gradual reduction thereafter [17]. Because maximal edema and lesion volume occur after hospital discharge, patients may experience more noticeable AEs during this period, which are subsequently reported at the two-week follow-up. Another group has attributed delayed speech changes to subtle alterations that emerge months after the procedure, potentially due to reorganization of cortical-subcortical networks affected by the lesion [18].

Long-term follow-up at two years identified only one patient with persistent speech deficits. This patient was also the only individual who experienced transient dysarthria during the initial procedure. Therefore, patients who experience speech-related AEs after the initial thalamotomy may be more likely to develop persistent speech difficulties following the second-side procedure. Future controlled studies are needed to confirm the role of intraoperative speech evaluation during bilateral MRgFUS thalamotomy. Although our study did not include a control group, previously published literature suggests that second-side thalamotomy is associated with a significant increase in speech-related AEs. This was not observed in our study. With the use of intraoperative speech evaluation, initial speech-related AEs were less frequent following the second-side procedure. Although the limitations of this study preclude confirmation of a causal relationship, our findings suggest a potential association between intraoperative speech evaluation and reduced initial speech changes following second-side MRgFUS VIM thalamotomy.

Limitations

Several limitations should be considered when interpreting this study. First, the sample size was relatively small (N = 13). This limited the statistical power of the study and precluded meaningful comparative and inferential statistical analyses. Staged bilateral MRgFUS thalamotomy for ET remains relatively uncommon in routine clinical practice. Assembling a larger cohort is even more challenging when comprehensive intraoperative speech evaluation is incorporated, as we are the first group, to our knowledge, to report this approach. Although larger studies have evaluated the safety and efficacy of MRgFUS thalamotomy, this study is unique in emphasizing the potential role of intraoperative speech evaluation in minimizing speech-related complications. Despite the limited sample size, this study provides valuable clinical insight into speech outcomes in this specialized patient population.

A second limitation is the retrospective nature of the study. Retrospective data collection limited the ability to establish control groups and standardize study variables. Nevertheless, this design provided an opportunity for an initial investigation before undertaking more focused prospective studies. Another limitation is that tremor outcomes and AEs were determined primarily through subjective patient reporting, with the exception of the formal intraoperative speech evaluation. Although reliance on subjective reporting may introduce reporting and recall bias, patient-reported outcomes are highly relevant because quality of life and functional impact are important patient-centered outcomes. Furthermore, the use of patient-reported outcomes is consistent with previous studies evaluating tremor control and AEs.

An additional limitation is the selective nature of the long-term follow-up. Two-year postoperative follow-up data were obtained only for the three patients who reported speech changes at the two-week follow-up. This methodological limitation may have introduced follow-up bias. Although unlikely, it is possible that patients who were not contacted developed delayed speech deficits, which would have affected the study findings. Nevertheless, this approach allowed assessment of symptom resolution in patients with speech-related AEs while avoiding unnecessary burden on asymptomatic patients.

Finally, this study lacked a control group. Although inclusion of a comparison group would strengthen the conclusions, the primary objective was to describe the clinical experience and the potential role of intraoperative speech evaluation in minimizing speech-related complications, thereby providing a foundation for future, more comprehensive investigations.

## Conclusions

Patient-reported outcomes were highly favorable, with the overwhelming majority indicating that the substantial improvement in tremor burden outweighed any temporary or mild AEs. These findings suggest that MRgFUS may offer a viable, less invasive alternative to traditional surgical approaches, such as DBS, for patients requiring bilateral tremor control. Initial speech-related AEs following second-side thalamotomy were limited when intraoperative speech evaluation was used. These findings suggest that incorporating intraoperative speech evaluation during second-side treatment may help minimize speech difficulties without compromising optimal targeting for maximal tremor control.
